# Two Homologues of the Global Regulator Csr/Rsm Redundantly Control Phaseolotoxin Biosynthesis and Virulence in the Plant Pathogen *Pseudomonas amygdali* pv. phaseolicola 1448A

**DOI:** 10.3390/microorganisms8101536

**Published:** 2020-10-06

**Authors:** Diana Ramírez-Zapata, Cayo Ramos, Selene Aguilera, Leire Bardaji, Marta Martínez-Gil, Jesús Murillo

**Affiliations:** 1 Institute for Multidisciplinary Research in Applied Biology, Universidad Pública de Navarra, 31192 Mutilva Baja, Spain; diana.ramirez@unavarra.es (D.R.-Z.); leire.bardaji.goikoetxea@gmail.com (L.B.); 2 Área de Genética, Facultad de Ciencias, Universidad de Málaga, Campus Teatinos s/n, E-29010 Málaga, Spain; crr@uma.es (C.R.); martamgv@uma.es (M.M.-G.); 3 Instituto de Hortofruticultura Subtropical y Mediterránea «La Mayora», Consejo Superior de Investigaciones Científicas (IHSM-UMA-CSIC), E-29010 Málaga, Spain; 4 Departamento de Química y Bioquímica, Instituto Tecnológico de Tepic, Colonia Lagos del Country, CP 63175 Tepic, Nayarit, Mexico; seleagui@gmail.com

**Keywords:** phaseolotoxin, phytotoxins, GacS/GacA system, two-component signal transduction system, small regulatory RNAs, *Pseudomonas syringae*, *Pseudomonas savastanoi*, virulence, post-transcriptional regulation

## Abstract

The widely conserved Csr/Rsm (carbon storage regulator/repressor of stationary-phase metabolites) post-transcriptional regulatory system controls diverse phenotypes involved in bacterial pathogenicity and virulence. Here we show that *Pseudomonas amygdali* pv. phaseolicola 1448A contains seven *rsm* genes, four of which are chromosomal. In RNAseq analyses, only *rsmE* was thermoregulated, with increased expression at 18 °C, whereas the antagonistic sRNAs *rsmX1*, *rsmX4*, *rsmX5* and *rsmZ* showed increased levels at 28 °C. Only double *rsmA*-*rsmE* mutants showed significantly altered phenotypes in functional analyses, being impaired for symptom elicitation in bean, including in planta growth, and for induction of the hypersensitive response in tobacco. Double mutants were also non-motile and were compromised for the utilization of different carbon sources. These phenotypes were accompanied by reduced mRNA levels of the type III secretion system regulatory genes *hrpL* and *hrpA*, and the flagellin gene, *fliC*. Biosynthesis of the phytotoxin phaseolotoxin by mutants in *rsmA* and *rsmE* was delayed, occurring only in older cultures, indicating that these *rsm* homologues act as inductors of toxin synthesis. Therefore, genes *rsmA* and *rsmE* act redundantly, although with a degree of specialization, to positively regulate diverse phenotypes involved in niche colonization. Additionally, our results suggest the existence of a regulatory molecule different from the Rsm proteins and dependent on the GacS/GacA (global activator of antibiotic and cyanide production) system, which causes the repression of phaseolotoxin biosynthesis at high temperatures.

## 1. Introduction

The ability of free-living bacteria to respond and adapt to environmental changes relies on various interconnected regulatory networks, from which the Csr/Rsm (carbon storage regulator/ repressor of stationary-phase metabolites) is one of the most intensively studied post-transcriptional regulatory systems [1,2,3]. This system is widely present and conserved among nearly 3,000 species of bacteria and act as a global regulator of gene expression of hundreds of genes including, among others, those supporting robust growth and a diverse collection of virulence genes in animal and plant pathogens.

The central component of the Csr/Rsm system is CsrA, which is also called RsmA and various other names in different bacteria [1,2]. For clarity, we will refer collectively to these proteins as Csr/Rsm proteins, and to the corresponding genes as *csr*/*rsm*, but in the main text we will use the proposed uniform nomenclature for the nine Rsm subfamilies for *Pseudomonas* [2]. Csr/Rsm is a small protein of 50-150 amino acids acting as a homodimer to bind RNAs at a 12 nt sequence containing a highly conserved GGA motif typically included in a stem-and-loop structure. Since its first detailed functional analysis [4], Csr/Rsm proteins have generally been associated to translational repression by a variety of mechanisms. Prominently, the Csr/Rsm binding site is often located close to and within the Shine–Dalgarno sequence, so protein binding prevents ribosomal interaction with the mRNA and often leads to destabilization of the downstream RNA. However, Csr/Rsm proteins can also mediate repression by favoring Rho-dependent transcription termination and, conversely, lead to gene activation by exposing the Shine–Dalgarno sequence upon binding to mRNA or by blocking its cleavage by RNase E [5]. The vast majority of the genomes examined contain a single copy of a *csr*/*rsm* gene. However, several bacterial lineages, particularly from *Legionella, Pseudomonas* and *Xanthomonas*, contain two to seven functional *csr*/*rsm* homologues, which are often carried by mobile genetic elements [2]. The homologues show variable levels of sequence identity, and within *Pseudomonas* can be classified into nine different protein subfamilies plus a group of unassigned proteins. Homologues of RsmA (CsrA2) and RsmE (CsrA3) are present in many, but not all bacteria containing a Csr/Rsm system; members of the other Rsm subfamilies, however, have a rather patchy distribution [2]. Although the exact role of the different homologues is often unclear, some of them were shown to be redundant and/or have unique regulatory roles, which likely provide genetic flexibility and helps to fine-tune the regulatory responses [6,7,8,9,10].

In *Gammaproteobacteria*, the activity of Csr/Rsm proteins is mainly modulated by various small untranslated regulatory RNAs (sRNA), among which RsmX, RsmY and RsmZ are the most relevant and intensely studied in pseudomonads [11]. The sRNAs modulating Csr/Rsm proteins are very diverse in length (approx. 100-479 nt) and predicted secondary structure, containing from 5 to 22 potential binding sites including the GGA motif. The sRNAs are thus molecular mimics that act as “protein sponges” [12], being able to sequester, store and release up to nine Csr/Rsm dimers [13], hence temporarily relieving their regulatory effect by competition with the mRNA target. In fact, they appear to be dedicated solely to sequestration of Csr/Rsm molecules. There is no correlation between the numbers of sRNAs and Csr/Rsm homologues in a given genome and, likewise, the sRNAs do not appear to be specific for each Csr/Rsm homologue [5]. Nevertheless, the sRNAs contribute to a differential control of the Csr/Rsm system because they show distinct affinities for different Csr/Rsm homologues and because they have distinct expression patterns, which can also change between different bacteria [10,14,15].

The levels of these small RNAs are in turn regulated by several factors, of which a main activator is the two-component signal transduction system GacS/GacA (global activator of antibiotic and cyanide production) [3,11]. This system was first described in *P. syringae* and *P. fluorescens* and shown to participate in virulence, ecological fitness, and antifungal activity, and is widely conserved among *Gammaproteobacteria* [2]. The GacS/GacA signaling is activated by various metabolites, including short-chain carboxylates and citrate, leading to autophosphorylation of the transmembrane sensor histidine kinase GacS, which in turn phosphorylates GacA for activation [1,3]. The only known targets of GacA are the genes for the antagonist sRNAs, and so it is assumed that the global regulatory effects of GacS/GacA is entirely mediated via the Csr/Rsm system [1,2]. Nevertheless, GacS/GacA activates most, but not all of the sRNAs antagonizing Csr/Rsm proteins, whereas Csr/Rsm homologues also interact with other global regulatory systems; therefore, there is not complete overlap between the Csr/Rsm and the GacS/GacA regulons. An important point is that, despite their widespread distribution in bacteria, the signals activating the GacS/GacA–Csr/Rsm systems and the target genes that are concomitantly regulated substantially differ among various bacteria, leading to large variations in the range and intensity of the phenotypes that are ultimately controlled [2,11].

*Pseudomonas syringae* sensu lato includes several gammaproteobacterial species that have been reassigned to at least six different genomospecies, mostly comprising plant pathogens, that are further subdivided into more than 60 pathovars according to characteristic plant host range [16]. *Pseudomonas amygdali* pv. phaseolicola (syn. *P. syringae* pv. phaseolicola and *P. savastanoi* pv. phaseolicola; Pph) belongs to genomospecies 2, comprising five previously named species and 26 pathovars of *P. syringae*. Pph causes economically significant epidemics of halo blight on bean (*Phaseolus vulgaris*) and mung bean (*Vigna radiata*) and is a prominent research model in plant pathology [17,18]. Pph is ubiquitous and causes severe yield losses in cooler regions (18 °C–22 °C), whereas species of *Xanthomonas* become the prevalent bacterial pathogens in warmer environments, indicating a particular adaptation of Pph to temperature to maximize fitness. Most strains of Pph produce the antimetabolite phytotoxin phaseolotoxin, which inhibits the biosynthesis of arginine and polyamines and leads to the chlorotic haloes typical of the disease [19,20]. Genes for the biosynthesis of the toxin are thermoregulated, with maximal expression at around 18 °C and negligible expression at 28 °C [21]. A DNA microarray analysis of Pph strain NPS3121 also identified many genes involved in pathogenicity and virulence that were potentially up-regulated at 18 °C [22]. Several pathogenicity and virulence genes depend on the GacS/GacA system in diverse strains of *P. syringae* sensu lato, although there are large variations between the phenotypes affected in different strains [23,24,25,26,27]. In Pph strain 1448A, the GacS/GacA system was found to contribute mildly to virulence by controlling gene *hrpL*, which is a master regulator for the expression of the type III secretion system (T3SS) and T3SS effector genes [28]. Importantly, the expression of phaseolotoxin biosynthesis genes appears to be subjected to post-transcriptional repression at 28 °C, because overexpression of *rsmY* in Pph NPS3121 led to phaseolotoxin biosynthesis at this non-permissive temperature [29,30]. In fact, the GacS/GacA system was shown to be essential for the expression of phaseolotoxin biosynthesis genes in strain NPS3121 [31], suggesting that this system might contribute to the thermoregulation of virulence genes. 

The roles of the Csr/Rsm system have only started to be investigated in strains of *P. syringae* sensu lato. *P. syringae* pv. tomato DC3000 contains five *csr*/*rsm* genes [9], and they were shown to contribute to the regulation of relevant roles for the bacterial life cycle, including the interaction with the plant host [9,10]. Homologues *rsmA* and *rsmE* (also called *csrA2*/*rsmA2* and *csrA3*/*rsmA3*, respectively) acted both individually and synergistically for the control of diverse phenotypes, displaying certain regulatory specificities, whereas no major roles could be assigned to the other homologues. However, the role of the Csr/Rsm system has not yet been explored in other members of *P. syringae* sensu lato.

In this work, we identified seven *rsm* gene homologues (*rsmA*, *rsmC*, *rsmE*, *rsmH1*, *rsmH2*, *rsmH3-1*, and *rsmH3-2*) in strain Pph 1448A. We generated mutants of this strain containing single and multiple mutations in its seven *rsm* genes, to evaluate their contribution to virulence and the thermoregulation of toxin genes, as well as to explore their role in regulating phenotypes that may be linked to the GacS/GacA system. Our results indicate that these seven *rsm* genes have a degree of specialization and a differential regulation, revealing the existence of variations in the Csr/Rsm regulatory circuitry with respect to *P. syringae* pv. tomato DC3000. We show that double mutants in genes *rsmA* and *rsmE*, but not individual mutants, show alterations in diverse phenotypes relevant for the life cycle of Pph 1448A, including virulence in bean, motility, metabolism of carbon sources and biosynthesis of the phytotoxin phaseolotoxin. 

## 2. Materials and Methods 

### 2.1. Bacterial Strains, Plasmids, and Growth Conditions

Bacterial strains and vectors used in this study are detailed in Table 1 and Table S1. *Escherichia coli* and *Pseudomonas* strains were routinely propagated at 37 °C and 25 °C, respectively, using either Luria-Bertani (LB) medium [32] or medium B [33]. When necessary, media were supplemented with (final concentrations, in µg mL^−1^): ampicillin, 100; gentamicin, 10; kanamycin, 25; spectinomycin, 25. 

### 2.2. Molecular Procedures

All primers (Table S2) were designed using the Primer3plus software [34]. DNA amplifications were performed with a standard enzyme (BIOTaq, Bioline, London, UK) or, for cloning, with a high-fidelity enzyme (PrimeStar HS, Takara Bio Inc., Kusatsu, Japan). Amplicons were purified using the PCR Extract Mini Kit (5 PRIME Inc.) when needed for sequencing (Macrogen Inc., Madrid, Spain) or for cloning using the CloneJET PCR Cloning Kit (Thermo Scientific, Vilnius, Lithuania). Plasmids were purified from *E. coli* employing a boiling method [35] or, for sequencing, a commercial kit (Illustra plasmidPrep Mini Spin Kit, GE Healthcare, Thermo Fisher Scientific SL, Madrid, Spain). Constructs were transferred to *P. syringae* by electroporation [36]. The integrity of all constructs was confirmed by sequencing.

For quantitative real-time reverse transcription PCR (RT-qPCR) analyses of *hrpL* and *hrpA* [38,39], bacterial strains grown overnight in LB at 25 °C were washed twice in the *hrp*-inducing minimal medium with fructose (MMF) [40], adjusted to an optical density at 600 nm (OD_600_) of 0.5 in the same medium and incubated with shaking (90 rpm) at 20 °C for 24 h before collecting the cells by centrifugation. For RT-qPCR of *fliC*, cells were grown on LB plates for 2 d at 18 °C or 28 °C. Plates were flooded with 1 mL of sterile distilled water, and cells were then resuspended and collected by centrifugation. Pelleted cultures were frozen in liquid nitrogen before RNA extraction.

RNA isolation was done using TriPure Isolation Reagent (Roche Diagnostics) and the Ambion TURBO DNA-free Kit (ThermoFisher Scientific, Vilnius, Lithuania), and cDNA was synthesized using random hexanucleotides (Promega, Madison, WI, USA) together with the ImProm-II reverse transcriptase system (Promega, Madison, WI, USA). qPCR experiments, using gene *gyrA* as reference, were carried out in the CX96TM Real-Time System and analyzed using the CFX Manager software version 3.0 (Bio-Rad Laboratories, Inc., Chicago, IL, USA), essentially as described in [38,39]. Gene expression levels were estimated using the ΔΔCt method [41] and the statistics were performed using R Project 3.3.3 [42].

For RNA-seq analyses, two independent biological replicates of Pph 1448A and gacA::IS mutant strains were grown in minimal standard succinate medium (SSM) [43], at 18 °C and 28 °C, until an OD_600_ of 0.7. Total RNA was extracted as above and its quality assessed on an Agilent Bioanalyzer 2100 using an RNA Pico 6000 chip (Agilent Technologies, Santa Clara, CA, USA). The two biological samples for each combination of strain and temperature treatment were sequenced and analyzed by the Servicios Centrales de Apoyo a la Investigación (SCAI) of the Universidad de Málaga (Spain). Raw reads were pre-processed using the SeqTrimNext pipeline [44] (http://www.scbi.uma.es/seqtrimnext) using the specific next-generation sequencing (NGS) technology configuration parameters, and the clean reads aligned with the closed genome of Pph 1448A (assembly ASM1220v1) with Bowtie 2 [45] in BAM (Binary Alignment/Map) files, which were then sorted and indexed using SAMtools v1.4 [46]. Differentially expressed genes between two samples were analyzed using the Tuxedo Tools (http://cole-trapnell-lab.github.io/cufflinks/tools/) [47]. The abundance of transcripts were measured in fragments per kilobase of fragments of gene per million reads (FPKM), and differentially expressed genes between two samples were analyzed using Cuffdiff [48] and considering a *p*-value < 0.05 as the significance threshold.

### 2.3. Bioinformatics Tools

Searches for *csr*/*rsm* homologues were done in the National Center for Biotechnology Information (NCBI) databases using the basic local alignment search tool (BLAST) algorithms. Sequence alignments were performed using the Multalin program [49] or the tools at the EMBL-EBI server (http://www.ebi.ac.uk/Tools/msa/), and construction of shaded alignments was done using the Sequence Manipulation Suite (http://www.bioinformatics.org/sms/) [50]. We used the MEGA7 software (v. 7.0.26) [51] for phylogenetic reconstructions, including multiple-sequence alignments with the MUSCLE program, determining the optimal substitution model, and construction of maximum-likelihood phylogenetic trees; confidence levels of the branching points were determined using 200 bootstraps replicates. Protein secondary structure was predicted using the JPred4 web server (http://www.compbio.dundee.ac.uk/jpred4/index.html) [52].

### 2.4. Mutagenesis and Cloning of rsm Genes

During the course of a random mutagenesis experiment of strain 1448A, using the minitransposon IS-Ω-Km/hah [53], we obtained mutants that did not produce phaseolotoxin and that contained insertions in genes *gacA* and *gacS* (positions 2,703,793 and 4,266,894, respectively, in genome accession no. CP000058), which were retained and designated as strains gacA::IS and gacS::IS, respectively.

Genes *rsmA*, *rsmC*, *rsmE* and *rsmH1* were mutagenized by marker exchange mutagenesis using appropriate DNA fragments or amplicons (see Table S2 for primers) cloned in vector pK18*mobsacB* [54] and using LB plus 5 % sucrose for counterselection. The DNA fragments from positions 1,745,318-1,746,973 and 3,778,776-3,779,364 (accession no. NC_005773), which include the complete *rsmC* and *rsmE* genes, respectively, were deleted from the chromosome. Gene *rsmA* was interrupted by inserting the Ω fragment (Sm^r^/Sp^r^) from pHP45Ω [55] extracted with BamHI, which is symmetrically bordered by stop codons in the three reading frames followed by a transcription termination signal, into its internal BclI site and truncating the deduced product after the first 13 amino acids. In turn, the reading frame of gene *rsmH1* was interrupted by filling-in its unique EcoRI site with Klenow (New England Biolabs, Ipswich, MA, USA), truncating the deduced product after the first 18 amino acids. We also used strain ΔpA (D. Ramírez-Zapata, unpublished results; Table 1), which derives from strain 1448A by curing of the native plasmid p1448A-A. This strain, therefore, lacks the plasmid-borne genes *rsmH2*, *rsmH3-1* and *rsmH3-2*. Multiple *rsm* mutants were constructed in a progressive manner, by the stepwise mutation of individual genes of strains 1448A or ΔpA as detailed above. For overexpression and complementation experiments, we individually cloned each of the *rsm* genes, plus between 0.19 and 0.5 kb of DNA preceding the annotated start codon, behind the *P*_BAD_ inducible promoter of the pJN105 expression vector [56]. In these clones, the *rsm* genes would be expressed from the native promoter and also, thanks to the already described leaky activity of the *P*_BAD_ promoter, from the vector [56]. Since we observed comparable results in media without and with 0.1 % arabinose, which leads to a high level of transcription from the *P*_BAD_ promoter [56], complementation and overexpression experiments were carried out using culture media supplemented only with gentamicin, to select for pJN105 clones. All mutants and clones in pJN105 were confirmed by PCR and sequencing.

### 2.5. Pathogenicity Assays and Autoagglutination

Plant growth and leaf inoculation were done essentially as described [57]. Bean (*Phaseolus vulgaris* L.) cultivar Canadian Wonder (CW) was grown in chambers at 23 °C–18 °C day–night temperatures, with a 16 h photoperiod and 70 % relative humidity. Bacteria freshly grown overnight on medium B plates were washed and suspended in ¼ Ringer’s (Oxoid, Basingstoke, UK) to an OD_600_ of 0.002 (approx. 10^6^ cfu mL^−1^) for inoculation of bean leaves. Tobacco plants (*Nicotiana tabacum* L. cv. Petit Havana) were held under the same photoperiod and humidity conditions, but the growing temperature was 28 °C and the cell suspensions for inoculation were adjusted to OD_600_ of 0.02 (approx. 10^7^ cfu mL^−1^). Cell suspensions were infiltrated into bean unifoliate leaves, or tobacco leaves, by piercing the abaxial surface with a needle and pressuring them through it, using a blunt syringe. Bean pods were obtained from a local supermarket (cv. Helda) or produced from locally grown plants (cv. Canadian Wonder) and were inoculated either using a toothpick [57] or by carefully injecting bacterial suspensions (approx. 10^7^ cfu mL^−1^) under the epidermal area [58], and scored daily for symptoms development. Bacterial populations in leaves were estimated essentially as described [59]. Briefly, two 0.6-cm diameter discs were harvested with a cork borer for each combination of date and inoculation replica and homogenized in 1 mL of ¼ Ringer’s. Serial dilutions of these suspensions were then plated on LB, to restrict growth and facilitate colony counting after 24–48 h of incubation at 25 °C. Nine replicates per strain were used for each of the three independent experiments performed on bean plants, and means were compared by an analysis of the variance (ANOVA *p* < 0.05). For tobacco, a total of at least twenty-seven inoculations per strain were performed in three independent experiments.

For Congo red staining and autoagglutination assays [60], strains grown on plates of medium B at 25 °C for 48 h were resuspended and adjusted to an OD_600_ of 0.5 in the *hrp*-inducing medium MMF [40]. Then, 5 µL of the resulting suspensions were deposited in the center of MMF plates supplemented with 20 µg mL^−1^ of Congo red, and were incubated at 20 °C for 48 h; additionally, 2 mL of each suspension were incubated with shaking (90 rpm) at 20 °C for 24 h before evaluating autoagglutination. All experiments were performed at least three times with three replicas each.

### 2.6. Assays of Motility and Biosynthesis of Phaseolotoxin

Swarming motility assays were done as described [9]. Briefly, bacteria grown on plates of medium B at 25 °C for 48 h were resuspended and adjusted to an OD_600_ of 2.0; then, 2 µL of the cell suspensions were deposited on semisolid peptone glucose agar (PG-agar) plates (0.5 % agar, 0.5 % proteose peptone Nº 3 and 0.2 % glucose). Plates were incubated at 18 °C or 28 °C for 48 h before photography.

Production of phaseolotoxin was assayed by an *E. coli* growth inhibition assay [61,62,63,64] with slight modifications. Briefly, a single colony of the indicator strain *E. coli* CECT 831 grown overnight on LB at 37 °C was used to inoculate 10 mL of LB and growth continued with shaking until an OD_600_ of 0.7, then centrifuged and resuspended in 3 mL of sterile distilled water. A 100 μL aliquot of this suspension was mixed with 3.5 mL of sterile 0.7 % agar in water supplemented with 100 μL of 20 % glucose and, when necessary, 100 μL of a 100 mM solution of appropriate amino acids, and spread over a plate of Ayer’s minimal medium [65]. Strains of *P. syringae* were grown at 18 °C or 28 °C in the Hoitink & Sinden optimized for coronatine production (HSC) minimal medium [66]. The different derivatives of strain 1448A, either mutants or strains overexpressing *rsm* genes, grew at slightly different rates in HSC; therefore, we evaluated the production of phaseolotoxin after 14-21 h, when the cultures were growing exponentially and had reached an OD_600_ of 0.2-0.3, and after 48 h of growth, in stationary phase (OD_600_ >1). At each time point, 20 µL of cell-free spent supernatants were deposited on 6 mm Whatman antibiotic assay discs arrayed on a sterile Petri dish cover; when ready, all the discs were transferred to the plate overlaid with the *E. coli* indicator strain and a further 10 μL of sterile distilled water was added to each disc to facilitate diffusion of the toxin into the medium. After 24–48 h of incubation at 37 °C, production of phaseolotoxin was confirmed by the appearance of growth-inhibition haloes on Ayer’s minimal medium plates that were inverted on plates with L-citrulline but not with L-ornithine.

All experiments were repeated at least three times with three replicas each.

### 2.7. Carbon Source Utilization

Bacterial strains grown for 2 d on LB plates at 25 °C were resuspended in ¼ Ringer’s to an OD_600_ of 0.1 (approx. 5 × 10^7^ cfu mL^−1^). These suspensions were used to inoculate microtiter plates to a final density of 5 × 10^6^ cfu mL^−1^ in a final volume of 150 µL, for 96-well plates, or 450 µL, for 48-well plates, of the appropriate culture medium. Plates were cultured for 72 to 96 h at 28 °C, with continuous shaking, in a multi-Detection Microplate Reader (Synergy^TM^ HT; Biotek^®^ Instruments, Winooski, VT, USA), which recorded OD_600_ reads every 30 min. As culture media we used either LB, SSM [43] or the minimum medium HSC [66]; for HSC, the original 20 g L^−1^ of glucose were substituted by 4 g L^−1^ of glucose, L-glutamine or L-glutamic acid. In each experiment we included three to six replicas for each combination of strain/culture medium, and experiments were repeated at least three times. 

## 3. Results and Discussion

### 3.1. Pseudomonas amygdali pv. phaseolicola Contains Seven rsm Homologues

**Figure 1:**
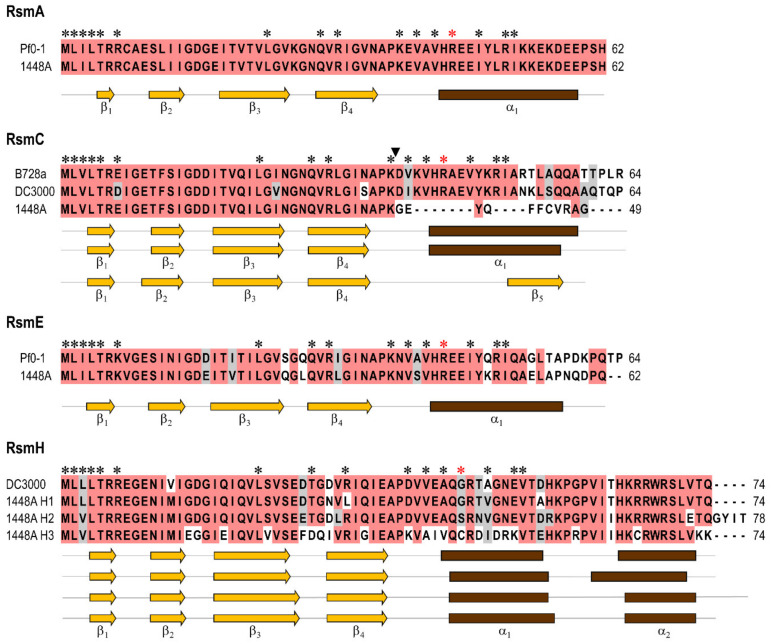
Sequence and structural conservation of the deduced products of the *rsm* homologues from *P. amygdali* pv. phaseolicola 1448A. All proteins were aligned using ClustalW, and identical or similar residues, shared by at least 60% of the sequences in multiple alignments, are shaded in red and grey, respectively. Dashes indicate gaps introduced to maximize the alignment. Asterisks indicate residues that are important for the interaction between RsmA or RsmE with RNA and for the regulation of several phenotypes [67,68,69], with the red asterisk indicating a critical Arg44 residue in RsmA and RsmE; the corresponding residues in RsmC and RsmH were marked from a sequence alignment with RsmA and RsmE. The black arrowhead indicates the point where an insertion of IS*Psy17* interrupts the *rsmC* coding sequence in strain Pph 1448A. Secondary structures were predicted with JPred4 and are shown below the alignments and in the same vertical order than the respective sequences; secondary structures were identical for RsmA and RsmE proteins, and so only one is shown for each. Abbreviations (accession numbers): Pf0-1, *P. fluorescens* Pf0-1 (RsmA, WP_002554426; RsmE, WP_003179932); 1448A, *P. amygdali* pv. phaseolicola 1448A; B728a, *P. syringae* pv. syringae B728a; DC3000, *P. syringae* pv. tomato DC3000; accession numbers for 1448A, B728a and DC3000 proteins are indicated in Table S3.

Pph 1448A contains seven *rsm* gene homologues (Figure 1 and Table S3). In order to maintain a consistent, coherent and uniform nomenclature across species, we adopted the recently proposed schema to designate them [2]. The genes encoding RsmA, RsmC, RsmE, and RsmH1 are located in the chromosome and three (*rsmH2*, and two identical copies of *rsmH3*) in the virulence plasmid p1448A-A. By comparison, *P. syringae* pv. tomato DC3000 contains five *rsm* gene homologues [9], although only the four chromosomal homologues of 1448A are conserved, with synteny and high identity, in strain DC3000. Additionally, strain DC3000 contains the chromosomally-encoded homologue RsmD (CsrA4) that is not present in strain 1448A (Figure S1 and Table S3).

The different protein homologues from strain 1448A are also similar among them, although they can be separated into four of the recently defined subfamilies within the genus *Pseudomonas* (Figure 1 and Figure S1). RsmA, RsmC and RsmE are frequent among pseudomonads and, in particular, RsmA and RsmE are highly conserved and the most commonly found, suggesting that they might regulate similar processes in this group of bacteria [2,9]. The other three homologues, RsmH1, RsmH2 and RsmH3, were assigned to the RsmH subfamily [2] because they cluster with high confidence with members of this group in a maximum likelihood tree and also contain a typical second alpha helix at the C-terminus (Figure 1 and Figure S1). These three proteins show a lower conservation of the amino acids that were found to be important for the interaction of RsmA and RsmE with RNA (Figure 1) [67,68,69]. Remarkably, RsmH1, RsmH2 and RsmH3 also show a lower degree of sequence conservation within *P. syringae* sensu lato (Figure S1) and also a very limited distribution among pseudomonads [2], suggesting that they might regulate phenotypes that are strain- or pathovar-specific. From this group, RsmH3 shows a more restricted distribution within *P. syringae* sensu lato.

**Figure 2:**
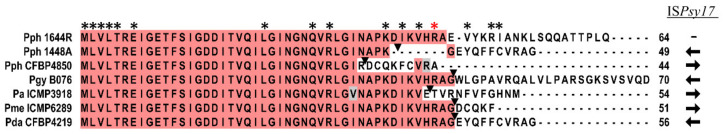
Disruption of gene *rsmC* by independent insertions of IS*Psy17* in diverse strains of genomospecies 2 of *Pseudomonas syringae* sensu lato. The deduced products of *rsmC* from the strains shown on the left were aligned using Multalin [49]. Pa, *P. amygdali*; Pda, *P. amygdali* pv. daphniphylli; Pgy, *P. amygdali* pv. glycinea; Pme, *P. meliae*; Pph, *P. amygdali* pv. phaseolicola. Only the first 70 residues are shown from the 116 residues sequence from Pgy B076. The first sequence, from strain 1644R, is a wild-type full-length allele; black triangles are inserted before the first amino acid modified by the insertion of IS*Psy17* and the arrow to the right indicates the orientation of this mobile element. Numbers at the end of the alignment indicate the total number of amino acids for each deduced product. Residues that are identical or similar in at least 50% of the sequences are shaded in red and grey, respectively. Asterisks indicate residues that are important for the interaction between the related proteins RsmA or RsmE with RNA and for the regulation of several phenotypes, with a red asterisk indicating the critical Arg44 residue [67,68,69].

RsmC in strain 1448A likely has a reduced or abolished activity because an insertion of IS*Psy17* interrupts and modifies the 3′ end of gene *rsmC*, shortening the deduced product from 64 to 49 amino acids (Figure 1 and Figure 2). Notably, the genomes of several other strains from genomospecies 2 also possess a mutated copy of *rsmC* interrupted with an insertion of IS*Psy17* (Figure 2). IS*Psy17* is by far the most abundant mobile element in strain 1448A and it appears to insert randomly [70]. In fact, the insertions of IS*Psy17* found in *rsmC* are in both orientations and in different positions, suggesting that they represent independent insertions rather than the result of target specificity. In all cases, the insertion eliminates amino acids that are important for the interaction between RsmA or RsmE with RNA (Figure 2), and for the regulation of several phenotypes in other bacteria [67,68,69]. In particular, the arginine residue at position 44 (Arg44) is critical for a strong binding to the RNA targets [69] and is missing in several of the insertional mutants. This is somewhat puzzling, however, because the Arg44 residue is also missing in RsmD (CsrA4) from strain DC3000 [9], and in RsmH1, RsmH2 and RsmH3 from strain 1448A (Figure 1). It is therefore possible that the stabilizing role of Arg44 is carried out by other positively-charged residues in these Rsm homologues (Figure 1). Alternatively, these homologues might in fact bind RNA with less efficiency and contribute to modulate the regulatory activity of the Csr/Rsm system, for instance by forming heterodimers with RsmA or RsmE and reducing or preventing their binding to RNA. Nevertheless, interruption of *rsmC* likely confers a selective advantage, given the repeated occurrence of IS*Psy17* insertions. However, it is not clear that these insertions will lead to a complete inactivation of the protein, or to changes in its specificity and/or the strength of its interactions. Therefore, it is possible that the regulatory activity of RsmC has a negative impact for the bacterial life cycle of diverse strains of genomospecies 2 and/or that the activity of the new RsmC product is advantageous.

**Figure 3:**
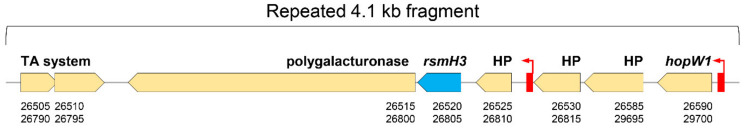
Gene *rsmH3* is located within a duplicated fragment in plasmid p1448A-A from *P. amygdali* pv. phaseolicola 1448A. Coding sequences are depicted as block arrows, with that of *rsmH3* highlighted in blue, and with numbers at the bottom indicating the PSPPH_RS locus tag for each of the two copies (accession no. NC_007274). TA system indicates a putative toxin–antitoxin system of the RelE/ParE family; polygalacturonase, is a glycoside hydrolase family 28 protein; HP, hypothetical protein; *hopW1* is a type III secretion system effector that contributes to the virulence of Pph 1448A [71]. The red boxes show the location and orientation of promoters regulated by HrpL (*hrp* boxes) [72].

Gene *rsmH3* lies within a 4,133 nt fragment (Figure 3) that is duplicated in plasmid p1448A-A [37]. This fragment also includes a polygalacturonase gene, immediately 3′ of *rsmH3*, and the T3SS effector virulence gene *hopW1* [71], and it is present with high Blastn identity in many pathovars of diverse genomospecies of *P. syringae* sensu lato. Additionally, gene *rsmH3* is preceded by a canonical *hrp* box [72], suggesting that it is part of the HrpL regulon and thus possibly involved in the post-transcriptional regulation of genes only during the interaction with plants. However, a previous RNA-seq analysis of the HrpL regulon did not evidenced expression of this gene [73].

### 3.2. Construction of Single and Multiple rsm Mutants

To evaluate the phenotypes regulated by the different *rsm* homologues, we constructed derivatives of Pph 1448A containing mutations in individual *rsm* genes as well as mutations in two or more of these genes. The chromosomal genes were individually mutated by marker exchange mutagenesis, either by complete deletion (*rsmC* and *rsmE*), by interrupting the coding sequence (CDS) with an antibiotic resistance cassette (*rsmA*), or by frameshifting, filling-in a restriction site (*rsmH1*). Strain ΔpA derives from strain 1448A and lacks the large plasmid (Ramírez-Zapata, unpublished results), consequently lacking *rsmH2* and both copies of *rsmH3*. Strains with multiple mutations were constructed from strains 1448A and ΔpA by multiple rounds of marker-exchange mutagenesis. For simplicity, all mutant strains will be henceforth designated with the gene(s) mutated in that strain (see Table 1).

To be used as controls, we obtained strains gacA::IS and gacS::IS by random transposon mutagenesis with the mobile element IS-Ω-Km/hah.

### 3.3. Expression Levels of rsm Genes

We evaluated by RNAseq the global transcription patterns of strains 1448A and gacA::IS during mid-exponential growth (OD_600_ of 0.7) in minimal medium at 18 °C and 28 °C. Here we report the expression values of *gacS*/*gacA*, the seven *rsm* homologues and the seven regulatory small RNAs, *rsmX1-5*, *rsmY* and *rsmZ* (Table 2).

As it occurs with *P. syringae* pv. syringae B728a [74], gene *gacA* transcription is not thermoregulated in strain 1448A. However, it is significantly less expressed at 28 °C in strain gacA::IS, suggesting that GacA stimulates its own transcription at high temperatures. Notably, expression of gene *uvrC* is nearly abolished in strain gacA::IS, likely due to a polar effect of the insertion of IS-Ω-Km/hah in *gacA*, as recently described for a *gacA*::Tn*5* insertion mutant of *P. syringae* pv. syringae B728a [75]. Therefore, the phenotypes displayed by strain gacA::IS in this work should be interpreted cautiously, because they might be due to defects in *gacA* and/or in *uvrC*. Nevertheless, the gacA::IS mutant is used in this work only as a control and to confirm previously reported phenotypes using similar mutants.

The *rsm* genes, except *rsmE*, showed levels of expression at 18 °C that were not significantly different from those at 28 °C. Gene *rsmE*, however, was significantly overexpressed at 18 °C. Most of the *rsm* genes, except *rsmE* and *rsmH3*, appear to not depend on GacA for their expression. The expression of *rsmE* was significantly reduced in strain gacA::IS at 18 °C compared to that of the wild-type (WT) strain, indicating that GacA acts as an activator of *rsmE* expression at 18 °C. Expression of the two copies of *rsmH3* in strain gacA::IS at 28 °C was significantly higher than in the wild-type strain. Since the expression of *rsmH2* was unchanged, however, this result indicates that changes in *rsmH3* transcription are not due to possible changes in plasmid copy number but rather to GacA likely mediating the repression of *rsmH3* at 28 °C.

Genes *rsmX1*, *rsmX2*, *rsmX3* and *rsmY* were on average expressed two- to eight-fold more than the other *rsm* sRNA genes both at 18 °C and 28 ºC (Table 2), suggesting that they might have a main role in gene regulation. Only four of the *rsm* genes (*rsmX1*, *rsmX4*, *rsmX5*, and *rsmZ*) appear to be thermoregulated, with significantly lower levels of expression at 18 ºC than at 28 °C. This suggests that they have a different role in the thermoregulation of gene expression in strain *P. amygdali* pv. phaseolicola 1448A. In *P. fluorescens* CHA0, genes *rsmX*, *rsmY* and *rsmZ* are also thermoregulated, although their expression is significantly higher at the lower temperature (30 °C vs. 35 °C) [76]. Additionally, and with the exception of *rsmZ* at 28 °C, all the sRNAs were significantly less expressed in strain gacA::IS suggesting that their expression is ultimately dependent on GacA. The GacS/GacA system has also been shown to activate the transcription of *rsmX* and *rsmY* in *P. amygdali* pv. tabaci [76], and of *rsmY* and *rsmZ* in *P. aeruginosa* [77] and *P. protegens* CHA0 [78], although with a differential regulation for *rsmZ* in strain CHA0 [78] and in *P. syringae* pv. *syringae* B728a [79]. This is relevant because *P. aeruginosa* also contains sRNAs antagonists of Rsm proteins that are independent of the GacS/GacA system [15,80].

### 3.4. Genes rsmA and rsmE Redundantly Control Virulence in Bean and the Expression of the T3SS

The GacS/GacA system regulates expression of pathogenicity or virulence in diverse pathovars of *P. syringae*. However, this system contributes only modestly to virulence of Pph 1448A in bean, through regulation of gene *hrpL* [28,81]. We therefore evaluated the role of the different Rsm homologues in the virulence of strain 1448A.

#### 3.4.1. Virulence in bean 

**Figure 4:**
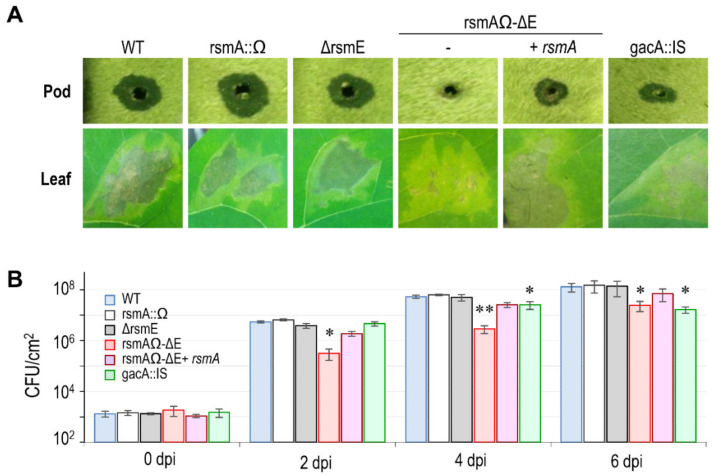
Genes *rsmA* and *rsmE* redundantly control virulence in bean (*Phaseolus vulgaris*). (**A**) Representative symptoms induced by *P. amygdali* pv. phaseolicola 1448A (WT) and derivative mutants in pods and leaves of bean cv. Canadian Wonder at 6 days post-inoculation. (**B**) Growth course of WT and mutant strains in leaves of bean cv. Canadian Wonder, after inoculation with suspensions of 10^6^ cfu mL^−1^. *; significant differences to WT, **; significant differences to WT and the *gacA* mutant, as determined by an ANOVA (*p* < 0.05). Error bars correspond to the standard deviation of three biological replicates, each with three technical replicates. Similar results were obtained for strain rsmAΩ-∆E complemented with *rsmA* or with *rsmE*.

As expected based upon [28], the *gacA* mutant of 1448A showed a moderately reduced virulence when inoculated on bean pods, causing repetitively smaller lesions than the wild-type strain, 1448A (Figure 4). Diverse combinations of single, double and multiple mutations of the *rsm* homologues did not have any apparent effect on the development of symptoms, except for those strains containing mutated versions of both *rsmA* and *rsmE*, which did not induce any water-soaking on bean pods (Figure 4A and data not shown). This suggests that only these two *rsm* genes are required, redundantly, for full virulence. Henceforth, we therefore concentrated on analyzing the role of only these two genes.

Inoculation of bean leaves produced similar results to that of bean pods, namely that strain gacA::IS and the double mutant *rsmA–rsmE* (rsmAΩ-∆E) induced less severe symptoms than the wild-type strain (Figure 4A). The reduction in symptoms expression was correlated with a significantly reduced ability to grow in planta (Figure 4B). Strain gacA::IS showed only a small, but significant reduction at 4 dpi, reaching population levels two to five times lower than the wild-type strain. This difference, however, increased at 6 dpi because populations of the wild-type strain, but not those of the *gacA* mutant, continued to increase. The defect in virulence was considerably more pronounced for strain rsmAΩ-∆E, which consistently reached population counts that were around one order of magnitude lower than those of the wild type at all the sampling times. Strains with a single mutation in either *rsmA* or *rsmE*, or complementation of strain rsmAΩ-∆E with either of these homologues, led to the induction of symptoms and to population levels similar to those displayed by the wild-type strain (Figure 4B and data not shown).

#### 3.4.2. Expression of the T3SS. 

The T3SS delivers specialized effector proteins into eukaryotic cells, facilitating pathogenicity and promoting virulence of many bacterial pathogens [81]. We evaluated the phenotypical and genotypical expression of the T3SS by examining the induction of the hypersensitive response (HR) in tobacco, autoagglutination and the expression of key regulatory genes. When inoculated at 10^7^ cfu mL^−1^, the wild-type strain and individual *rsmA*, *rsmE* and *gacA* mutants all produced a similar HR reaction in tobacco leaves within 24×48 h, whereas strain rsmAΩ-∆E did not induce an HR in the majority of the inoculations, even after 2 d (Figure S2). The elicitation of the HR in some of the inoculated points is likely due to a partial activation of the T3SS and could also be influenced by local population levels and leaf age. In particular, partial defects of the T3SS and small variations in inoculated population levels were found to have a dramatic effect on the elicitation of the HR in diverse mutants of *P. syringae* pv. tomato DC300 [82]. Complementation of strain rsmAΩ-∆E with the individual *rsmA* or *rsmE* genes led to only a partial recovery of the phenotype. This type of partial complementation, or complementation to the reverse phenotype, has already been reported for *rsm* homologues in *P. syringae* pv. tomato DC3000 [9,10], again suggesting the relevance of the relative concentration of Rsm proteins in the cell. 

**Figure 5:**
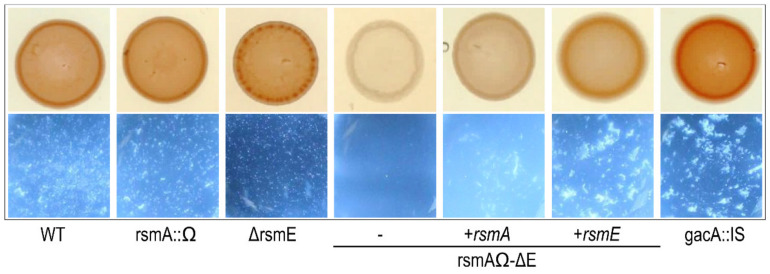
The double mutant *rsmA–rsmE* does not agglutinate in culture. Hrp-pilus production assay in *hrp*-induction medium by Congo red staining on plates (top) and autoagglutination in liquid (bottom). Pictures are representative of three independent experiments.

Staining of bacterial colonies on Congo red plates and autoagglutination in liquid medium were shown to correlate with the production of the Hrp pilus [60]. All strains, except strain rsmAΩ-∆E, stained red and were autoagglutination positive (Figure 5). This suggests that the biosynthesis of functional T3SS pili is compromised in the double mutant, likely explaining its reduced ability to induce the HR in tobacco. Remarkably, cultures of strains gacA::IS and rsmAΩ-∆E complemented with *rsmE* consistently showed many large cell clumps (Figure 5), likely suggesting a higher expression of the T3SS in these strains.

**Figure 6:**
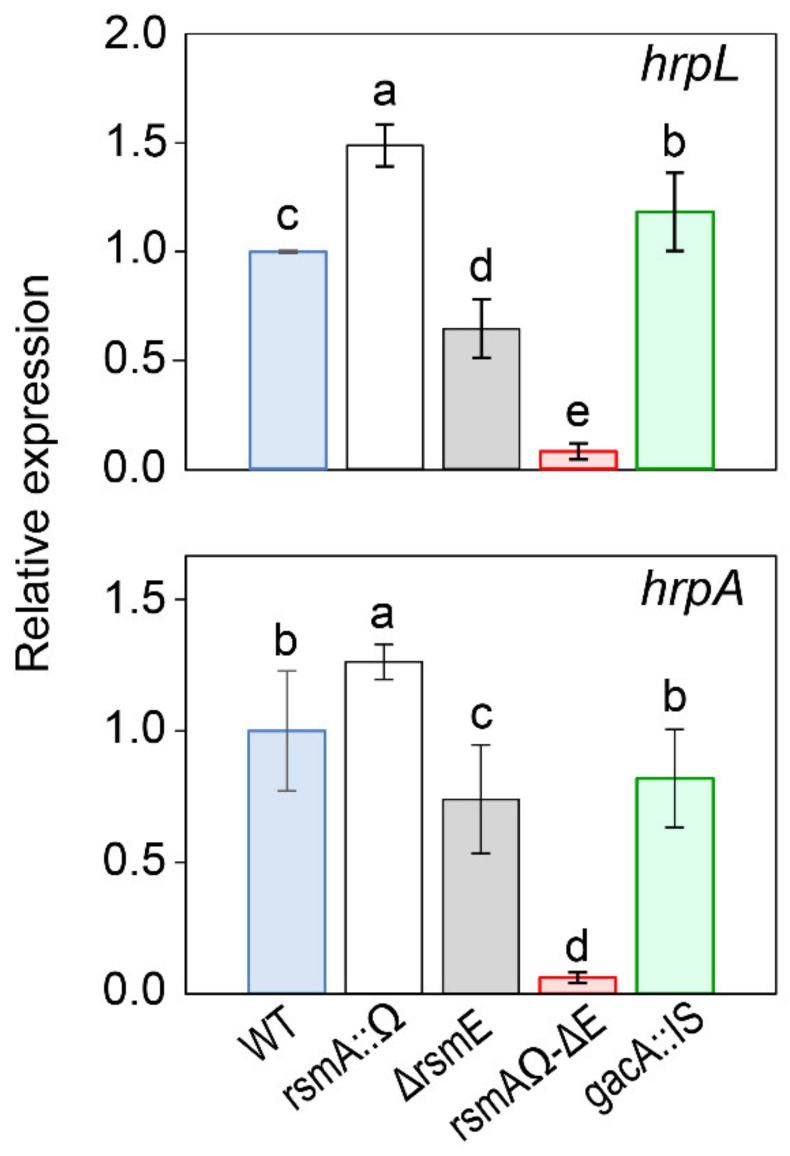
Expression analysis of type III secretion genes in *P. amygdali* pv. phaseolicola 1448A and derivative mutants. Bars indicate the expression of the indicated genes relative to strain 1448A (WT), after normalization with gene *gyrA* as an internal control, using quantitative real-time reverse transcription PCR (RT-qPCR). Letters above bars denote ANOVA categories with significant differences (*p* < 0.05). Error bars correspond to the standard deviation of three biological replicates.

We also examined the expression of the T3SS by measuring the relative expression levels of genes *hrpL* and *hrpA* by RT-qPCR (Figure 6). HrpL is an alternative sigma factor activating the expression of the *hrp* cluster and other genes essential for pathogenicity, whereas HrpA is the Hrp pilus protein, responsible for the delivery of effectors to plant cells and also a regulator of the T3SS expression [17,72,81]. Both genes *hrpL* and *hrpA* showed a higher level of transcription in the rsmAΩ mutant to the wild-type strain, but this was reversed in mutant ΔrsmE, with a significantly reduced transcription (Figure 6). Nevertheless, the reduced transcription level of strain ΔrsmE was sufficient to induce an HR on tobacco and to display apparently normal Congo red staining and autoagglutination phenotypes (Figure 5 and Figure S2). Strain rsmAΩ-∆E showed a further reduced level of transcription of *hrpL* and *hrpA*, agreeing with its severely reduced ability to cause the HR on tobacco and its lack of autoagglutination (Figure 5 and Figure S2). Therefore, RsmA and RsmE show a degree of specialization for the regulation of the biosynthesis of the T3SS. Thus, RsmA is dispensable for the expression of genes regulating the T3SS and can only partially substitute the activity of RsmE.

Together, our results indicate that the lower virulence of strain rsmAΩ-∆E and its inability to cause and HR in tobacco are likely due to a reduced expression of the T3SS and, probably because of a reduced expression of *hrpL*, of the T3SS effector genes. The partial functional redundancy of homologues of RsmA and RsmE has already been reported for the regulation of biocontrol factors in *P. fluorescens* CHA0 and in *P. syringae* pv. tomato DC3000, where they play a major role in virulence [9,10,11]. The role of RsmA and RsmE in regulating the T3SS in strain DC3000 is not entirely clear, however, with reports indicating highly increased or highly reduced transcription of *hrpL* and *hrpA* in individual *rsmE* mutants [9,10]. Additionally, and contrarily to strain rsmAΩ-∆E (Figure S2), a *rsmA*–*rsmE* double mutant of strain DC3000 was still able to induce the HR in tobacco like the wild-type strain [10]. This suggests that, in parallel with their high sequence conservation [2,9], the functionality of RsmA and RsmE is also in general widely conserved but might display certain strain-specific variations.

### 3.5. Genes rsmA and rsmE also Redundantly Control Motility and Carbon Source Utilization

We tested the involvement of *gacA* and *rsm* genes in motility and carbon source utilization, because these processes are part of the GacS/GacA regulon in a large diversity of bacteria and are particularly relevant for plant infection [3,11]. 

#### 3.5.1. Motility 

**Figure 7:**
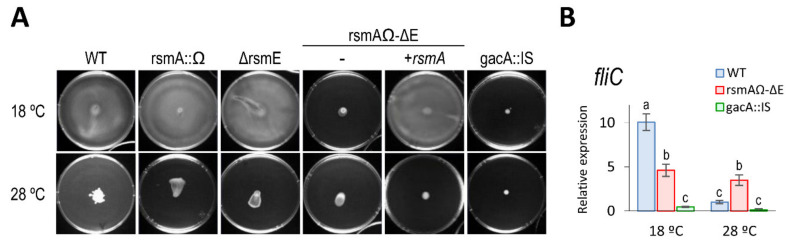
Swarming motility is thermoregulated and redundantly dependent on RsmA and RsmE in *P. amygdali* pv. phaseolicola 1448A. (**A**) Essay of motility at different temperatures. Strains were grown on the surface of peptone-glucose agar (0.5 %) for 48 h. Similar results were obtained for strain rsmAΩ-∆E complemented with *rsmA* or with *rsmE*. Pictures are representative of three replicates in three independent experiments. (**B**) Relative expression of the flagellin gene, *fliC*, assessed by quantitative real-time reverse transcription PCR (RT-qPCR). Bars represent the average expression relative to strain 1448A (WT) at 28 °C, after normalization with gene *gyrA* as an internal control. Letters above bars denote ANOVA categories with significant differences (*p* < 0.05). Error bars correspond to the standard deviation of three biological replicates.

Genes involved in motility are upregulated at 18 °C in *P. amygdali* pv. phaseolicola NPS3121, although this strain is non motile for unknown reasons [22,83]. As expected, therefore, strain 1448A showed high swarming motility at 18 °C but very low or not at all at 28 °C (Figure 7A). Reduced motility at 28 °C was correlated with a significant reduced transcription of gene *fliC* (Figure 7B), coding for the flagellin subunits that constitute the flagellum, which in turn is required for swarming motility [74]. 

Mirroring the case with other bacteria [3,9,84], the *gacA* mutant of 1448A was non motile in both conditions (Figure 7). This same phenotype was shown by strain rsmAΩ-∆E, but not by the corresponding single mutants. Swarming motility of the double mutant was complemented at 18 °C by gene *rsmA* or *rsmE* to wild-type levels (Figure 7A and data not shown). Strain Mut-7-rsm, containing no functional *rsm* genes, was non motile and was also complemented by either gene *rsmA* or *rsmE* (data not shown), indicating that they might be the only *rsm* genes regulating motility. Additionally, the motility defect of the *gacA* mutant and the double *rsmA*–*rsmE* mutant correlated with a downregulation of gene *fliC* (Figure 7B).

These results indicate that thermoregulation of motility in Pph 1448A involves the redundant activation of the flagellar apparatus at low temperature by RsmA and RsmE. In turn, the lack of expression of motility in the *gacA* mutant suggests the existence of a putative repressor that will be likely antagonized through the GacS/GacA system. In *P. syringae* pv. tomato DC3000, motility is also dependent on the redundant regulation by RsmA and RsmE at 20 °C [10].

#### 3.5.2. Carbon Source Utilization 

Strain 1448A showed diauxic growth in the rich medium LB (Figure S3), exemplified by two exponential growth phases separated by a small lag phase. Strain rsmAΩ-∆E, but not the individual mutants, showed instead a single exponential phase that, unexpectedly, allowed the reaching of higher optical densities. The diauxic growth is generally interpreted as an adaptation for the sequential metabolism of different carbon sources [85]; therefore, the double mutant is likely unable to efficiently metabolize one or more of the carbon sources available in the medium and is consequently growing on a single carbon source. The double *rsmA*–*rsmE* mutant, but again not the individual mutants, also showed a reduced and delayed growth in minimal media containing glucose, glutamic acid, glutamine, or succinate as the sole carbon source (Figure S3). In all cases, except for glucose, the growth defects were complemented by either *rsmA* or *rsmE* (data not shown).

The Csr/Rsm system, and the antagonist sRNAs, are involved in the regulation of genes involved in carbon flux pathways in numerous bacterial strains [5,14]. Pph 1448A is not an exception, and the metabolism of preferred carbon sources [86] is under the redundant control of RsmA and RsmE. Likewise, the metabolism of diverse favored carbon sources is also under the control of the Csr/Rsm system. The regulation network, however, seems to be very complicated because individual mutations in *rsmE* already led to a significant growth reduction with diverse carbon sources in strain DC3000 [9,10].

### 3.6. RsmA and RsmE Redundantly Contribute to the Activation of Phaseolotoxin Biosynthesis at 18 °C

Three lines of evidence would suggest that an Rsm protein might be involved as a repressor in the thermoregulated biosynthesis of phaseolotoxin. First, production of phaseolotoxin is abolished in a strain of Pph overexpressing *rsmA* from *P. aeruginosa* PAO1 [83]. Second, a *gacA* mutant of Pph is unable to synthesize the toxin [31]; such a mutant will be unable to express the sRNAs antagonizing RsmA [5], which will predictably cause the full repression of the Pht cluster. Third, a small DNA fragment containing the *rsmY* gene allows for the biosynthesis of phaseolotoxin at 28 °C when present in multiple copies, likely by titrating a repressor [29,30]. It is likely that this repressor might be an Rsm protein, because *rsmY* is known to sequester Csr/Rsm proteins [30].

#### 3.6.1. Toxin Biosynthesis by Strain 1448A Depends on GacA and GacS

**Figure 8:**
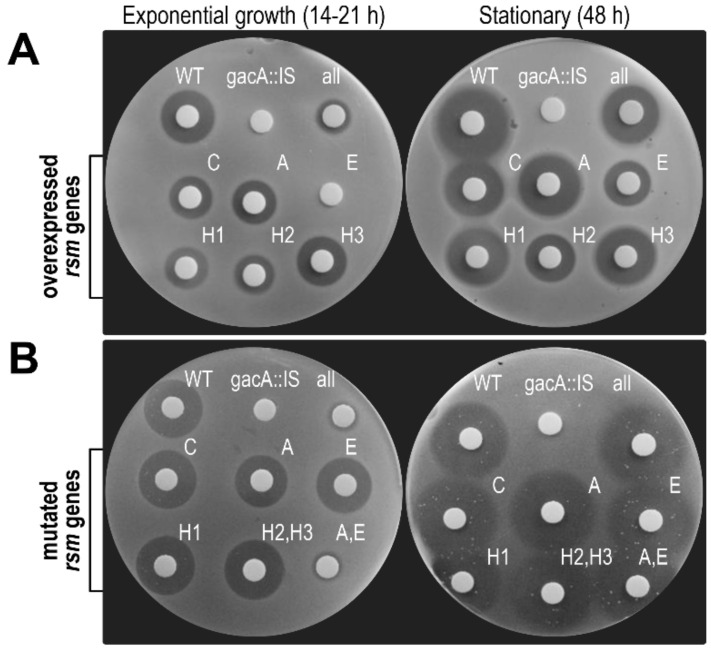
Differential role of the Rsm homologues on phaseolotoxin biosynthesis. (**A**) Biosynthesis of phaseolotoxin by derivatives of *P. amygdali* pv. phaseolicola 1448A overexpressing different *rsm* genes or (**B**) containing mutations inactivating one, two or all (all, strain Mut-7-rsm) of the seven *rsm* homologues. WT, wild type strain 1448A; gacA::IS, a *gacA*-minus transposon mutant of strain 1448A. Strain labeled as H2,H3 (strain ΔpA) is lacking alleles *rsmH2* and the two copies of *rsmH3*. Toxin production was evidenced as haloes of growth inhibition in an *E. coli* indicator assay. Pictures are representative of three independent experiments.

Strains gacA::IS and gacS::IS were unable to synthesize phaseolotoxin (Figure 8 and data not shown), as it occurs with strain Pph NPS3121 [31]. This indicates that regulation of phaseolotoxin biosynthesis is similar in both strains 1448A and NPS3121. Although phenotypes shown by insertional mutants in *gacA* might be due to polar effects on *uvrC* [75], abolition of phaseolotoxin biosynthesis in strain gacS::IS indicates that the corresponding genes are controlled by the GacS/GacA system in strain 1448A. 

#### 3.6.2. Overexpression of rsmE Suppresses Phaseolotoxin Synthesis only in Exponentially Growing Cultures 

To confirm that the Rsm products repress the expression of the Tox cluster, and to disclose any possible specificity, we overexpressed the different *rsm* homologues in strain 1448A. We separately cloned each of the homologues with sufficient upstream sequences in pJN105 [56]; in this vector, they could be expressed from their own promoter, or highly overexpressed from the regulatable P*_BAD_* promoter of pJN105 by induction with arabinose. After transferring the clones to strain 1448A, we evaluated the biosynthesis of phaseolotoxin in media with and without arabinose, obtaining similar results in both cases (data not shown).

Overexpression of *rsmE* completely suppressed the production of phaseolotoxin by strain 1448A at 18 °C at early stages of growth (approx. 14–21 h; OD_600_ of 0.2–0.3) (Figure 8A). This repression, however, was partially alleviated in cultures grown for approximately 48 h (OD_600_ > 1). A possible increased level of *rsmY* expression with increasing cell density, which was shown to occur in *P. fluorescens* and *P. syringae* pv. tomato [30,76], might contribute to sequestration of Rsm proteins in stationary phase and likely participate in the repression alleviation. In turn, homologues *rsmC*, *rsmH1* and *rsmH2* repetitively lead to a small decrease in the production of phaseolotoxin. Additionally, we did not observed biosynthesis of phaseolotoxin at 28 °C by any of the strains and all haloes were reverted by supplementation with citrulline but not with ornithine (data not shown); since phaseolotoxin specifically blocks the conversion of ornithine to citrulline [20], this indicates that the haloes were produced by phaseolotoxin. These results agree with a previous report that overexpression of *rsmA* from *P. aeruginosa* PAO1 in Pph NPS3121 abolished production of phaseolotoxin [83], suggesting that RsmE, and RsmC, RsmH1 and RsmH2 to a minor degree, might repress toxin biosynthesis. However, they are somewhat unexpected because RsmA from Pph 1448A shows a very high identity to RsmA from strain PAO1 (accession no. NP_249596; 80.7 % id.), but had no activity as repressor (Figure 8A), whereas RsmE is less similar (63.5% id.) but leads to a strong repression (Figure 8A).

#### 3.6.3. Double rsmA–rsmE Mutants are Unable to Synthesize Phaseolotoxin only in Exponentially Growing Cultures 

To further examine their role, we also evaluated the biosynthesis of phaseolotoxin by strains with mutations in one or more *rsm* homologues (Figure 8B). We observed no significant variation in the amount of phaseolotoxin synthesized at 18 °C by strains containing only one defective *rsm* homologue or with different combinations of mutations in *rsmC*, *rsmH1*, *rsmH2* and *rsmH3* (Figure 8 and data not shown). In turn, derivatives lacking both homologues *rsmA* and *rsmE* showed a marked reduction in the production of the toxin in early stages of growth (Figure 8B). However, supernatants from stationary cultures produced inhibition haloes that were indistinguishable from those produced by the wild-type strain. A strain lacking the seven *rsm* homologues showed the same phenotype to strain rsmAΩ-∆E. Finally, there was no production of phaseolotoxin by the wild-type strain or any of the *rsm* mutants at 28 °C (data not shown). This regulatory activity resembles that for the biosynthesis of the phytotoxin coronatine by *P. syringae* pv. tomato DC3000 [10]. In strain DC3000, individual mutations in genes *rsmA* and *rsmE* reduced the expression of genes for the biosynthesis of coronatine, which was further reduced in a double *rsmA*–*rsmE* mutant, whereas gene *rsmD* (*csrA4*) has a minor role in regulation that is only noticeable in the absence of *rsmA* and *rsmE*. It is therefore feasible that the biosynthesis gene clusters for other phytotoxins that also depend on the GacS/GacA system in *P. syringae*, such as syringomycins [87], mangotoxin [64], or tabtoxin [88], are also redundantly induced by RsmA and RsmE.

Previous results [83] and our own data from overexpression experiments (Figure 8A) suggest that RsmE, and to a lesser extent RsmC, RsmH1, and RsmH2, might act as repressors of phaseolotoxin biosynthesis. However, this is in potential conflict with results from the analysis of mutant strains (Figure 8B) indicating the opposite effect, with a role of RsmA and RsmE as activators of toxin synthesis. This behavior mirrors the opposing phenotypes observed from overexpression or mutation of *rsmA* in *P. aeruginosa* PAO1 [89] or of *rsmA* and *rsmE* in *P. syringae* pv. tomato DC3000 [9,10], and further suggests that the cellular concentrations of the Csr/Rsm proteins are critical for proper regulation. The effects seen from gene overexpression might therefore be artefactual because of an abnormally high concentration of the regulator. Among other possible effects, this could lead to non-specific interactions or de-stabilization of the relative concentrations of the different molecules involved in regulation, for instance by titration of sRNAs targeting Rsm or other proteins.

In summary, our results indicate a differential role of the Rsm homologues on the regulation of phaseolotoxin biosynthesis in strain 1448A, with RsmA and RsmE redundantly activating it at 18 °C, at least in the early stages of growth. These proteins are however not essential, and derivative strains of 1448A containing diverse combinations of mutations or lacking all seven *rsm* gene homologues were still able to synthesize phaseolotoxin in a thermoregulated way (Figure 8). Therefore, thermoregulation of phaseolotoxin biosynthesis in Pph 1448A should depend on an as yet unidentified molecule, whose activity appears to be also dependent on the GacS/GacA system because a *gacA* mutant of Pph 1448A does not produce phaseolotoxin (Figure 8) [31]. This putative molecule is likely a repressor of phaseolotoxin biosynthesis because possession by Pph of multiple copies of *rsmY* allowed for phaseolotoxin biosynthesis at 28 °C [29,30]. The existence of such regulatory molecule is likely, because there are several bacterial species that possess the GacS/GacA system but no *csr/rsm* genes [2], suggesting that in these cases the GacS/GacA-mediated gene regulation will likely depend on other regulatory molecule(s).

## 4. Conclusions

*Pseudomonas amygdali* pv. phaseolicola 1448A contains seven *rsm* homologues, four of which (*rsmA*, *rsmC*, *rsmE*, and *rsmH1*) are chromosomal and three (*rsmH2* and two copies of *rsmH3*) are located in the virulence plasmid p1448A-A. Our results indicate that these genes have a degree of specialization and a differential regulation. Gene *rsmC* might have a detrimental activity for diverse bacteria from genomospecies 2 (*P. amygdali*), including strain 1448A, because their *rsmC* copies contain independent insertions of IS*Psy17* in the 3′ end that eliminate key residues for protein activity. An RNA-seq analysis indicates that homologues *rsmA*, *rsmC*, *rsmH1*, and *rsmH2* are not thermoregulated and that GacA does not influence their transcription rate. Conversely, mRNA levels of gene *rsmE* are significantly higher at 18 °C. The expression of *rsmE* and of the seven sRNA genes of strain 1448A at 18 °C appear to be dependent on GacA, because their mRNA levels were significantly reduced at 18 °C in a *gacA* mutant. Regulation of sRNAs, however, is complex, because the expression of *rsmZ*, but not of the other sRNAs, did not depend on GacA at 28 °C; additionally, only *rsmX1*, *rsmX4*, *rsmX5* and *rsmZ* showed thermoregulation, with increased mRNA levels at 28 °C.

In functional assays with strains containing mutations in one or more *rsm* homologues, we could associate altered phenotypes only to strains with mutations in both *rsmA* and *rsmE*. A double *rsmA-rsmE* mutant did not induce the typical water-soaking in detached bean pods and elicited only mild symptoms in bean leaves, reaching bacterial populations that were around one order of magnitude lower than the wild-type strain. This reduced virulence is likely due, at least in part, to the reduced mRNA levels in the mutant of genes *hrpL* and *hrpA*, which are key regulators of the expression of the type III secretion system [81]. The double mutant was non-motile and showed abnormal growth patterns in diverse culture media. RsmA (CsrA2) and RsmE (CsrA3) also play similar regulatory roles in *P. syringae* pv. tomato DC3000, probably derived from their high sequence conservation among pseudomonads [9,10]. Nevertheless, there appear to be strain-specific adaptations in their regulatory behavior. In particular, RsmA and RsmE exert non-redundant functions in strain DC3000, with individual mutations in *rsmE* having a significant impact in virulence, motility and carbon source utilization, among other phenotypes. In strain 1448A, however, we were able to detect changes in these phenotypes only in strains with mutations in both *rsmA* and *rsmE* genes.

Finally, our results show that Rsm proteins are not repressors of the biosynthesis of phaseolotoxin. Rather, RsmA and RsmE redundantly induce the biosynthesis of this phytotoxin at 18 °C. However, the corresponding genes are not essential, and a double *rsmA–rsmE* mutant is still able to synthesize phaseolotoxin at 18 °C, although only in cultures reaching high population densities. Biosynthesis of the toxin by 1448A is dependent on GacA and is thermoregulated, even in a derivative strain with no functional *rsm* genes. Therefore, these results suggest that the biosynthesis of phaseolotoxin is repressed at 28 °C by a yet unidentified molecule. This putative molecule is apparently integrated into the GacS/GacA regulatory system and has previously been shown to be antagonized by overexpression of the sRNA *rsmY* [29].

## Figures and Tables

**Table 1:** Relevant *Pseudomonas amygdali* pv. phaseolicola strains used in this study.

Strains	Main features^a^	Reference or source
1448A	Wild type (WT) strain, isolated from *Phaseolus* in Ethiopia, 1985	[37]
ΔpA	UPN1162; 1448A cured of plasmid p1448A-A; ∆*rsmH2* ∆*rsmH3-1* ∆*rsmH3-2*	D. Ramírez-Zapata, unpublished
∆rsmE	UPN1168; 1448A ∆*rsmE*	This work
ΔrsmC	UPN1187; 1448A ∆*rsmC*	This work
rsmA::Ω	UPN1225; 1448A *rsmA*::Ω	This work
rsmAΩ-∆E	UPN1227; derives from UPN1168 *rsmA*::Ω ∆*rsmE*	This work
Mut-7-rsm	UPN1229; derives from UPN1185 ∆*rsmC rsmA*::Ω ∆*rsmE rsmH1*-fsX ∆*rsmH2* ∆*rsmH3-1* ∆*rsmH3-2*	This work
gacA::IS	UPN1230; 1448A *gacA*::IS-Ω-Km/hah	This work
gacS::IS	UPN1362; 1448A *gacS*::IS-Ω-Km/hah	This work

^a^ UPN, collection number in the Universidad Pública de Navarra. Ω specifies insertion of the Ω fragment from pHP45Ω in the indicated gene. Gene *rsmH1*-fsX contains a filled-in EcoRI restriction site, introducing a frameshift (fsX) after position 54 of its coding sequence. See text and Table S1 for strain UPN1185 and for further details.

**Table 2:** RNAseq expression values of genes from the GacS/GacA and Csr/Rsm transcriptional regulation systems from *P. amygdali* pv. phaseolicola 1448A.

	FPKM^a^				Fold change (log_2_)^b^			
	WT		gacA::IS		18 °C/28 °C		gacA::IS/WT	
Gene	18 °C	28 °C	18 °C	28 °C	WT	gacA::IS	18 °C	28 °C
*gacS*	81.3	71.5	44.1	40.9	0.2	0.1	−0.9	−0.8
*gacA*	347.3	354.7	200.3	144.9	0.0	0.5	0.8	**−1.3**
*uvrC*	115.3	140.1	3.4	4.5	−0.3	−0.4	**−5.1**	**−5.0**
*rsmA*	932.9	565.1	1235.0	844.0	0.7	0.6	0.4	0.6
*rsmC*	53.8	75.1	67.1	43.9	−0.5	−0.6	−0.3	0.2
*rsmE*	614.6	241.7	179.2	204.9	**1.4**	−0.2	**−1.8**	0.2
*rsmH1*	179.8	228.2	168.2	280.5	−0.3	−0.7	−0.1	0.3
*rsmH2*	88.1	193.2	93.6	155.0	−1.1	−0.7	0.1	−0.3
*rsmH3-1*	96.2	169.5	86.2	419.2	−0.8	**−2.3**	−0.2	**1.3**
*rsmH3-2*	99.6	171.4	86.5	412.0	−0.8	**−2.3**	−0.2	**1.3**
**sRNAs**								
*rsmX1*	61440.0	181313.0	980.2	2283.9	**−1.6**	**−1.2**	**−6.0**	**−6.3**
*rsmX2*	85990.4	129285.0	765.7	1228.2	−0.6	−0.7	**−6.8**	**−6.7**
*rsmX3*	54830.1	51529.8	116.8	400.4	0.1	**−1.8**	**−8.9**	**−7.0**
*rsmX4*	338.7	737.1	71.4	100.1	−1.1	−0.5	**−2.3**	**−2.9**
*rsmX5*	682.1	2853.5	32.6	26.0	−2.1	0.3	**−4.4**	**−6.8**
*rsmY*	15372.1	11471.4	330.3	966.0	0.4	**−1.6**	**−5.5**	**−3.6**
*rsmZ*	791.7	2504.5	357.5	2712.2	**−1.7**	**−2.9**	**−1.2**	0.1

**^a>^** FPKM normalized, FPKM; fragments per kilobase of gene fragments per million readings. **^b^** Fold change (FC) indicates averaged differential gene expression (log_2_ normalized) in *P. amygdali* pv. phaseolicola 1448A (WT), or the derivative *gacA* mutant with respect to temperature or to each other; positive and negative fold changes reflect an increased or a decreased level of gene expression, respectively, at 18 °C or in the gacA::IS strain. Cells with grey shading indicate genes with a significant differential expression (*p*-value < 0.05).

## Supplementary Materials

The following are available online at https://www.mdpi.com/2076-2607/8/10/1536/s1. Figure S1: Phylogeny of Rsm proteins from selected strains of *Pseudomonas syringae* sensu lato and assignation to subfamilies, Figure S2: Effect of different *rsm* homologues from *P. amygdali* pv. phaseolicola 1448A on the elicitation of the hypersensitive response on tobacco leaves, Figure S3: Growth curve of *P. amygdali* pv. phaseolicola 1448A and derivative mutants in LB or minimal media with different carbon sources, Table S1: Bacterial strains and plasmids used in this study, Table S2: List and application of primers used in this work, Table S3: Characteristics of the *rsm* genes from *Pseudomonas amygdali* pv. phaseolicola 1448A and their products, with homologues in *P. syringae* pv. tomato DC3000 and *P. syringae* pv. syringae B728a.
