# Supplementary material for: Two Homologues of the Global Regulator Csr/Rsm Redundantly Control Phaseolotoxin Biosynthesis and Virulence in the Plant Pathogen Pseudomonas amygdali pv. phaseolicola 1448A
Source: Microorganisms. 2020 Oct 6;8(10):1536. doi: 10.3390/microorganisms8101536 (PMC7600136; doi:10.3390/microorganisms8101536)
Supplement: Supplementary file 1 [file microorganisms-08-01536-s001.pdf]

microorganisms

MDPI

Article

Two Homologues of the Global Regulator Csr/Rsm Redundantly Control Phaseolotoxin Biosynthesis and Virulence in the Plant Pathogen *Pseudomonas amygdali* pv. phaseolicola 1448A

Diana Ramírez-Zapata [1], Cayo Ramos [2,3], Selene Aguilera [4], Leire Bardaji [1], Marta Martínez-Gil [2,3] and Jesús Murillo [2,*]

[1] Institute for Multidisciplinary Research in Applied Biology, Universidad Pública de Navarra, 31192 Mutilva Baja, Spain; diana.ramirez@unavarra.es (D.R.-Z); leire.bardaji.goikoetxea@gmail.com (L.B.); jesus.murillo@unavarra.es (J.M.; ORCID 0000-0001-6585-2421)

[2] Área de Genética, Facultad de Ciencias, Universidad de Málaga, Campus Teatinos s/n, E-29010 Málaga, Spain; crr@uma.es (C.R.; ORCID 0000-0002-2362-5041); martamgv@uma.es (M.M.-G.)

[3] Área de Genética, Instituto de Hortofruticultura Subtropical y Mediterránea «La Mayora», Consejo Superior de Investigaciones Científicas (IHSM-UMA-CSIC), E-29010 Málaga, Spain

[4] Departamento de Química y Bioquímica, Instituto Tecnológico de Tepic, Colonia Lagos del Country, CP 63175 Tepic, Nayarit, México; seleagui@gmail.com

* Correspondence: jesus.murillo@unavarra.es; Tel.: +34-948168007

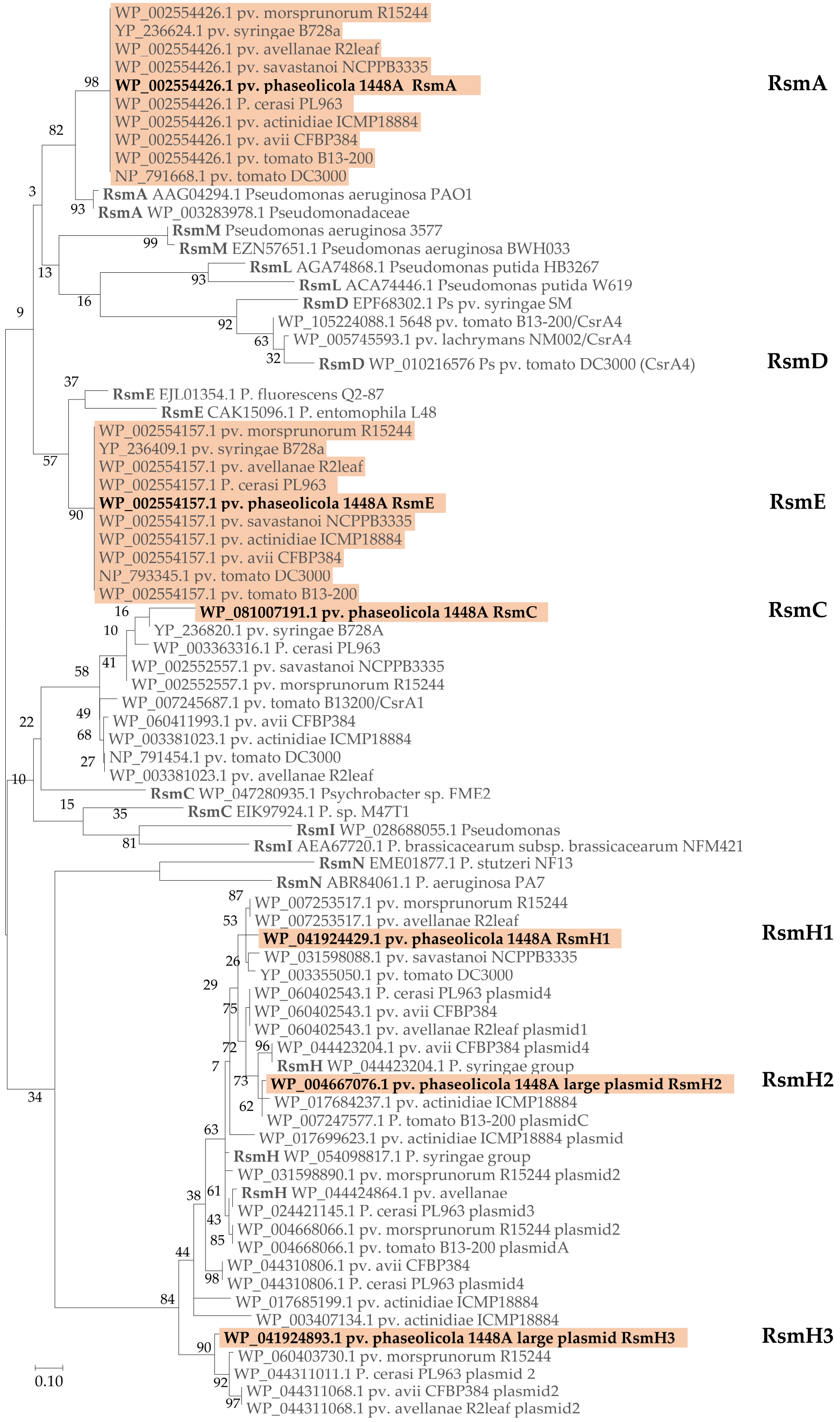

Figure S1. Phylogeny of Rsm proteins from selected strains of *Pseudomonas syringae sensu lato* and assignation to subfamilies. The Rsm proteins present in each genome were identified by Blastp comparison using as query the Rsm proteins from Pph 1448A and RsmD from *P.s.* pv. tomato DC3000; all the identified proteins were included to construct the tree plus two or three representative proteins from each of the 9 Rsm subfamilies [1]. For simplicity, strains previously classified as *P. syringae* are indicated only by their pathovar and strain assignation. Protein alignment with Muscle, identification of the best model and construction of the maximum likelihood tree, using the JTT model with a discrete Gamma distribution with five categories and using all sites, were done with MEGA7 [2]. Orange highlighting, proteins from strain Pph 1448A or identical to them in each clade. The tree is drawn to scale, with branch lengths measured in the number of substitutions per site. Numbers in branches indicate per cent bootstrap values with 200 replicates. To the right, name of the previously defined phylogenetic Rsm subfamilies [1].

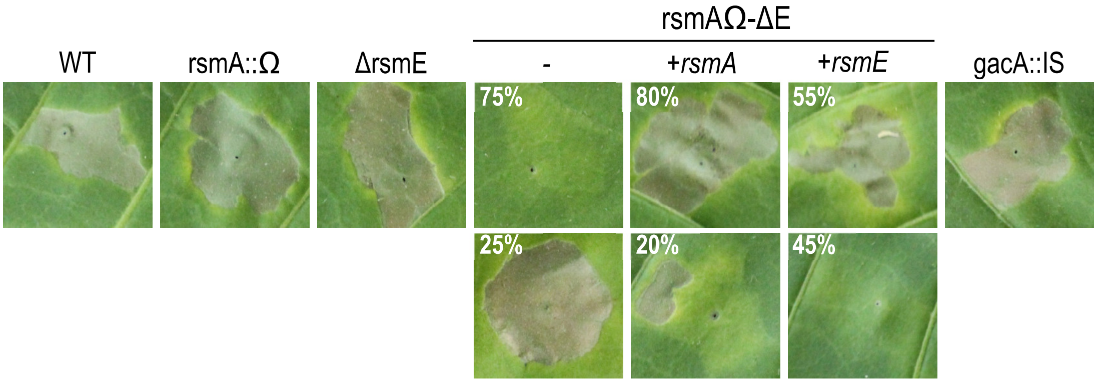

Figure S2. Effect of different *rsm* homologues from *P. amygdali* pv. phaseolicola 1448A on the elicitation of the hypersensitive response on tobacco leaves. Results are representative from at least 25 inoculations on at least four different plants. For mutants inducing different types of reaction, it is shown the percentage of each type.

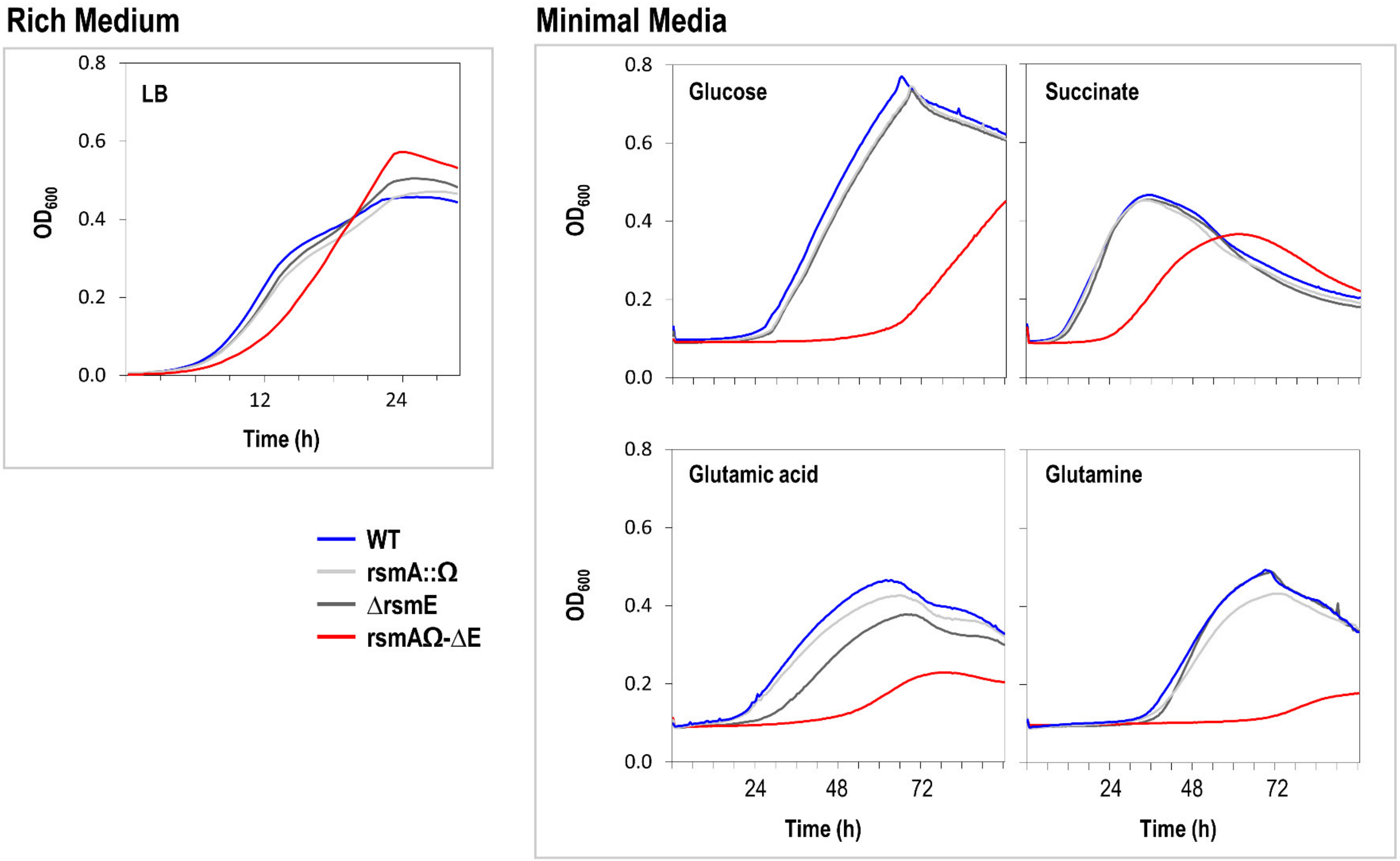

Figure S3. Growth curve of *P. amygdali* pv. phaseolicola 1448A and derivative mutants in LB or minimal media with different carbon sources. Cells were inoculated at 5 x 10^6 cfu/mL (for LB) or 5 x 10^7 cfu/mL (for minimal media) in 48-well plates, and incubated at 28 °C with continuous shaking in the multi-Detection Microplate Reader (Synergy HT; Biotek Instruments, Winooski, VT, USA), which recorded OD_{600} reads every 30 minutes. Each value is the average from three to six technical replicates. Carbon sources were added at 0.4 % (w/v) to HSC base minimal medium; for succinate, cells were grown in minimal medium SSM. Graphs are from one representative experiment out of three with similar results.

Table S1. Bacterial strains and plasmids used in this study[a]

Strain/plasmid	Main features[b]	Reference or source
	**Strains**	
	Escherichia coli	
NEB10β	Δ(*mrr-hsdRMS-mcrB*) *deoR recA1 endA1 araD139* Δ(*ara, leu*)7697 *galU galK λ⁻ rpsL nupG*	[3]
CECT831	Strain sensitive to phaseolotoxin, used as indicator for phaseolotoxin production bioassays	Colección Española de Cultivos Tipo (CECT)
	***Pseudomonas amygdali* pv. phaseolicola**	
UPN1160	1448A *rsmH1*-fsX	This work
UPN1164	Derives from UPN1162; *rsmH1*-fsX Δ*rsmH2* Δ*rsmH3-1* Δ*rsmH3-2*	This work
UPN1166	Derives from UPN1162; Δ*rsmE* Δ*rsmH2* Δ*rsmH3-1* Δ*rsmH3-2*	This work
UPN1176	Derives from UPN1168; Δ*rsmE rsmH1*-fsX	This work
UPN1184	Derives from UPN1176; Δ*rsmC* Δ*rsmE rsmH1*-fsX	This work
UPN1185	Derives from UPN1166; Δ*rsmC* Δ*rsmE* Δ*rsmH2* Δ*rsmH3-1* Δ*rsmH3-2*	This work
UPN1186	Derives from UPN1162; Δ*rsmE rsmH1*-fsX- Δ*rsmH2* Δ*rsmH3-1* Δ*rsmH3-2*	This work
UPN1226	Derives from UPN1162; *rsmA*::Ω Δ*rsmH2* Δ*rsmH3-1* Δ*rsmH3-2*	This work
UPN1228	Derives from UPN1166 Δ*rsmE rsmA*::Ω Δ*rsmH2* Δ*rsmH3-1* Δ*rsmH3-2*	This work
	**Plasmids**	
pSCR001	10,571 bp, GenBank accession no. DQ059989, carrying the minitransposon IS-Ω-Km/hah; Km^R	[4]
pHP45Ω	Broad-host-range plasmid, source of the Ω fragment (Sp^R/Sm^R)	[5]
pJET1.2	*E. coli* cloning vector 2.9 kb, Amp^R	Thermo Fisher Scientific
pJN105	Broad-host-range expression vector, carries the *L*-arabinose-inducible *E. coli araBAD* promoter and the *araC* regulator; Gm^R	[6]
pJNA1	pJN105 with a 533 pb fragment containing the *rsmC* gene flanked by EcoRI and SacI restriction sites; Gm^R	This work
pJNA2	pJN105 with a 488 pb fragment containing the *rsmA* gene flanked by EcoRI and SacI restriction sites; Gm^R	This work
pJNA3	pJN105 with a 1118 pb fragment containing the *rsmE* gene flanked by EcoRI and SacI restriction sites; Gm^R	This work
pJNA5	pJN105 with a 583 pb fragment containing the *rsmH1* gene flanked by PstI and SacI restriction sites; Gm^R	This work
pJNA6	pJN105 with a 723 pb fragment containing the *rsmH2* gene flanked by PstI and SacI restriction sites; Gm^R	This work
pJNA7	pJN105 with a 689 pb fragment containing the *rsmH3* gene flanked by EcoRI and SacI restriction sites; Gm^R	This work
pK18*mobsacB*	Mobilizable cloning vector, confers sucrose-dependent lethality; Km^R, Suc^S	[7]

[a] See Table 1 in the main text for other relevant strains. [b] Abbreviations: Amp, ampicillin; Km, kanamycin; Gm, gentamicin; Sp, spectinomycin; Suc, sucrose. Superscripts R and S denote resistance or susceptibility, respectively. Letter Ω specifies insertion of the Ω fragment in the indicated gene (see plasmid pHP45Ω for details).

Table S2. List and application of primers used in this work.

Primer name and purpose	Sequence (5′→3′)[a]	5′position[b]	Remarks
Construction of *csrA* mutations			
rsmC_F_ext	TGCCGACTACATCTTCAGGC	1,744,292	Complete deletion of *rsmC* and IS*Psy17*, from 1745318-1746971
rsmC-left_R	actagtCAGTAACGCCCTTCAAACGC	1,745,317*	
rsmC-right_F	actagtCGACGTTGTTCGCCTCATTG	1,746,972	
rsmC-right_R	actagtCGTCGATCTGTTGTTTCCGC	1,748,633*	
rsmA_R-mut	ATGTTCACCACTCTCGGCTG	4,045,687	For insertion of the Ω fragment into the unique BclI site of *rsmA*; truncates the product after amino acid 13, position 1,882,019
rsmA_F-mut	GCAAATACAACGTCCCGCTG	4,047,554*	
rsmE-left_F	GGTGTTCATTGTTGCCCGTC	3,777,563	Complete deletion of *rsmE*, from 3,778,776-3,779,364
rsmE-left_R	actagtTCTCGGCATGAGTGTAAGCG	3,778,775*	
rsmE-right_F	actagtTAATTGCTGCCTAACCCGCC	3,779,365	
rsmE-right_R	actagtCCTTTGGAAAACTCGGCAGC	3,780,522*	
rsmH1_F-mut	CACTGTAGCAGTACGGGGAC	894,653	To fill-in the unique EcoRI site in *rsmH1*, truncating the product after amino acid 18; position 895,777
rsmH1_R-mut	CTTTCGATCAACCACGCAGC	896,615*	
Cloning of *csrA* genes for overexpression and complementation			
rsmC_1504_F	gaattcTATGCAGGACGAGCCTATG	1,745,054	
rsmC_1504_R	gagctcCCAGACCGAGCTGGTAAAAC	1,745,567*	
rsmA_3510_R	gagctcACCCTTTTCCCCGTTTGC	4,046,581	
rsmA_3510_F	gaattcCCGTGGTCATCGAAGAGAAG	4,047,068*	
rsmE_3260 _F	gaattcGCTCAGATCAACCCGATCAT	3,778,194	
rsmE_3260 _R	gagctcGCCAGTAAATGGCAAATCAA	3,779,311*	
rsmH1_0763bis_ F	ctgcagCCAAACGTAAAGTCGCACTG	895,401	
rsmH1_0763bis _R	gagctcTTGTGCTCCTGATCTGGTTG	895,962*	
rsmH2_A0105 _F	ctgcagGGTGCTGTGCCAGAAATACC	91,582*	
rsmH2_A0105 _R	gagctcTACTGCGCGGCTATGTAATG	90,879	
rsmH3_A0073_F	gaattcCACGAGAAAAGACAGGTCCAC	5,618*	
rsmH3_A0073_R	gagctcAGCGAAATACCCACGGAAG	61,720	
Real time quantitative PCR			
gyrA_F	CGAGCTGAAGCAGTCCTACC	4,214,791*	
gyrA_R	CGGATTTCTTGTACGGCTTG	4,214,637	

qhrpL_F	GCGCAACGAGCACAAGTTT	1,505,838	
qhrpL_R	GGTTCAACGCAATACCACACAA	1,505,905*	
qhrpA_F	CAGGGTATCAACAGCGTCAAGA	1,487,675	
qhrpA_R	GCTACCCGTGTTTTTGGTCAGT	1,487,740*	
qhrpR_F	CCCGAATCGTTGGCAGAA	1,485,848	
qhrpR_R	CGAGCGCGGCAGACA	1,485,918*	
qFliC_F	TCCGCCAGCACCATGACTTTCC	3,920,267*	
qFliC_R	TCACAGCCGAACCGACACCC	3,920,174	
Other purposes			
pk18mob_R	caggaaacagctatgaca		Analysis of inserts

[a] Restriction sites introduced in primers to facilitate cloning are in underlined lowercase. [b] The coordinates of the annealing point of the first nucleotide of each primer is indicated. Unless otherwise indicated in the remarks column, coordinates are for the genome of strain 1448A (accession no. NC_005773). Asterisks indicate that the primer anneals in the reverse strand.

Table S3. Characteristics of the *rsm* genes from *Pseudomonas amygdali* pv. phaseolicola 1448A and their products, with homologues in *P. syringae* pv. tomato DC3000 and *P. syringae* pv. syringae B728a

Gene[a]	nt	aa	Locus tag	Old locus tag	Other names in Pto DC3000[b]	Protein accession number in *P. amygdali*/*P. syringae* pathovars		
						phaseolicola 1448A	tomato DC3000	syringae B728a
rsmA	189	62	PSPPH_RS17770	PSPPH_3510	*csrA2*/*rsmA2*	WP_002554426	WP_002554426	WP_002554426
rsmC	150	49	PSPPH_RS28320	PSPPH_1504	*csrA1*/*rsmA1*	WP_081007191	WP_003381023	WP_003402536
rsmD	-[c]	-	-	-	*csrA4*/*rsmA4*	-	WP_010216576	-
rsmE	189	62	PSPPH_RS16510	PSPPH_3260	*csrA3*/*rsmA3*	WP_002554157	WP_002554157	WP_002554157
rsmH1	225	74	PSPPH_RS03905	-[d]	*csrA5*/*rsmA5*	WP_041924429	WP_011103313	-
rsmH2[e]	237	78	PSPPH_RS26950	PSPPH_A0105	-	WP_004667076	-	-
rsmH3[e]	225	74	PSPPH_RS26520<br>PSPPH_RS26805	PSPPH_A0007<br>PSPPH_A0073	-	WP_041924893	-	-

[a] Rsm subfamilies as described [1]; genes were assigned to the RsmH subfamily because they clustered closely to RsmH-type proteins in a maximum likelihood tree reconstructed using sequences and methods described in Sobrero and Valverde [1] (see Supplementary Figure S1). [b] As designated previously [8,9]. [c] -, not present. [d] This gene was not annotated in the earlier versions of the genome. [e] Genes *rsmH2* and *rsmH3* are located in plasmid p1448A-A; *rsmH3* is present in two identical copies, within a 4,133 nt repeated fragment.

References

1. Sobrero, P.M.; Valverde, C. Comparative genomics and evolutionary analysis of RNA-binding proteins of the CsrA family in the genus *Pseudomonas*. *Front. Mol. Biosci.* **2020**, *7*, 127, doi:10.3389/fmolb.2020.00127.
2. Kumar, S.; Stecher, G.; Tamura, K. MEGA7: Molecular Evolutionary Genetics Analysis version 7.0 for bigger datasets. *Mol. Biol. Evol.* **2016**, *33*, 1870–1874, doi:10.1093/molbev/msw054.
3. Grant, S.G.; Jessee, J.; Bloom, F.R.; Hanahan, D. Differential plasmid rescue from transgenic mouse DNAs into *Escherichia coli* methylation-restriction mutants. *Proc. Natl. Acad. Sci. USA* **1990**, *87*, 4645–4649, doi:10.1073/pnas.87.12.4645.
4. Giddens, S.R.; Jackson, R.W.; Moon, C.D.; Jacobs, M.A.; Zhang, X.-X.; Gehrig, S.M.; Rainey, P.B. Mutational activation of niche-specific genes provides insight into regulatory networks and bacterial function in a complex environment. *Proc. Natl. Acad. Sci.* **2007**, *104*, 18247–18252, doi:10.1073/pnas.0706739104.
5. Prentki, P.; Krisch, H.M. *In vitro* insertional mutagenesis with a selectable DNA fragment. *Gene* **1984**, *29*, 303–313, doi:10.1016/0378-1119(84)90059-3.
6. Newman, J.R.; Fuqua, C. Broad-host-range expression vectors that carry the L-arabinose-inducible *Escherichia coli araBAD* promoter and the *araC* regulator. *Gene* **1999**, *227*, 197–203, doi:10.1016/S0378-1119(98)00601-5.
7. Schäfer, A.; Tauch, A.; Jager, W.; Kalinowski, J.; Thierbach, G.; Pühler, A. Small mobilizable multipurpose cloning vectors derived from the *Escherichia coli* plasmids pK18 and pK19: selection of defined deletions in the chromosome of *Corynebacterium glutamicum*. *Gene* **1994**, *145*, 69–73, doi:10.1016/0378-1119(94)90324-7.
8. Ferreiro, M.D.; Nogales, J.; Farias, G.A.; Olmedilla, A.; Sanjuan, J.; Gallegos, M.T. Multiple CsrA proteins control key virulence traits in *Pseudomonas syringae* pv. *tomato* DC3000. *Mol. Plant-Microbe Interact.* **2018**, *31*, 525–536, doi:10.1094/mpmi-09-17-0232-r.
9. Ge, Y.; Lee, J.H.; Liu, J.; Yang, H.-w.; Tian, Y.; Hu, B.; Zhao, Y. Homologues of the RNA binding protein RsmA in *Pseudomonas syringae* pv. tomato DC3000 exhibit distinct binding affinities with non-coding small RNAs and have distinct roles in virulence. *Mol. Plant Pathol.* **2019**, *20*, 1217–1236, doi:10.1111/mpp.12823.
